# PADA: perturbation-aware differential Riemannian attention for robust motor imagery decoding

**DOI:** 10.3389/fnins.2026.1943934

**Published:** 2026-09-24

**Authors:** Jianxiu Li, Luao Sha, Xiaokai Yan, Chunya Wang, Jiaxin Shi, Huiyu Qiao, Changming Wang, Yongfeng Wei, Caili Gong

**Affiliations:** 1 School of Electronic Information Engineering, Inner Mongolia University, Hohhot, China; 2 Inner Mongolia Key Laboratory of Intelligent Communication and Sensing and Signal Processing, Hohhot, China; 3 Department of Neurosurgery, Capital Medical University, Beijing, China

**Keywords:** differential attention, motor imagery, perturbation perception, Riemannian manifold, robustness

## Abstract

**Introduction:**

Riemannian geometry models have significantly improved the robustness and performance of EEG-based motor imagery decoding and are better suited to small sample sizes; however, their ability to extract nonlinear and spatiotemporal variability features often falls short of Euclidean-based deep learning methods. To address these limitations, we introduce a perturbation-aware differential Riemannian attention mechanism (PADA).

**Methods:**

PADA develops a window-based learnable Riemannian module that maps informative segments onto the manifold to characterize nonlinear features while preserving local structure, together with a two-stream differential manifold attention mechanism. Tangent-space perturbations are used as a probe for feature instability, and attention weights that are hypersensitive to noise are differentially suppressed to distill robust geometric dependencies.

**Results:**

On the BCI Competition IV-2a and Zhou2016 datasets, PADA achieved competitive clean-condition accuracies of 81.44% and 92.79%, respectively. Under 50% noise, its accuracy dropped by only 12%, achieving the best classification performance among the compared models. On a self-constructed dataset involving 227 subjects, PADA achieved up to 70.46% binary classification accuracy on the classic hand motor imagery task using only 12 training samples.

**Discussion:**

These results indicate that PADA provides robust and effective feature learning for noisy and data-limited brain–computer interface applications.

## 1 Introduction

Motor Imagery (MI) based on non-invasive Electroencephalography (EEG) has emerged as an important research direction owing to its low cost, high temporal resolution, and portability, offering broad application prospects in aiding motor function rehabilitation (Meng et al., 2025; Bai, 2025) and improving intelligent human-computer interaction (Hwaidi and Ghanem, 2026; Luo et al., 2025). Motor imagery refers to the mental simulation of a movement without any physical execution, a covert cognitive process that engages neural networks similar to those activated during actual motor performance (Jeannerod, 2001; Qu, 2014). However, EEG signals face many challenges in motor imagery classification tasks due to their low signal-to-noise ratio, non-stationarity, and individual variability.

To mitigate these challenges, deep learning models such as DeepConvNet and EEGNet have improved classification performance by automatically learning spatiotemporal features (Xue et al., 2024; Ying et al., 2025; Zhang et al., 2023). However, EEG signals are inherently characterized by a non-Euclidean geometry, with their spatial covariance matrices forming points on a symmetric positive definite (SPD) Riemannian manifold (Chen et al., 2026; Tibermacine et al., 2024). The local statistics of these signals exhibit complex nonlinear dependencies on the spatial distribution of electrodes (Tibermacine et al., 2024; Pan et al., 2023). Existing deep learning methods are fundamentally confined to euclidean space, making it difficult to effectively model the manifold structure of EEG signals. This structural contradiction has become a key bottleneck limiting the robustness of current deep learning models, which perform poorly in the face of inter-subject variability (Manivannan and Lakshminarayan, 2025).

As a compelling alternative, Riemannian geometry methods offer a promising solution by directly performing feature representation and classification on the SPD manifold. (Barachant et al., 2012) leveraged the SPD covariance matrices of EEG signals and harnessed the inherent robustness of Riemannian distances and means to propose the minimum distance to Riemannian mean classifier and tangent space LDA, achieving more stable BCI decoding. Based on this, classification methods based on the riemannian mean of the covariance matrix have been widely adopted. These methods classify an unknown sample by computing its riemannian distance to the riemannian mean point of each class, demonstrating robust performance in EEG signal decoding (Chevallier et al., 2021; Pan et al., 2025). (Jin et al., 2023) further integrated riemann-derived spatio-temporal covariance features with sparse optimization, utilizing dempster-shafer theory to aggregate classification results across multiple time windows, thereby significantly enhancing the decoding robustness of MI-BCI. By preserving the intrinsic geometric structure of the covariance matrix, riemannian geometry methods offer significant advantages in characterizing EEG nonlinear features and addressing non-stationarity. However, conventional riemannian methods suffer from complex iterative computation and considerable performance degradation in high-variability scenarios such as cross-subject or cross-session classification (Zhuo et al., 2024).

In response to the above limitations, a growing body of research has emerged that integrates riemannian geometry with deep learning. Specifically, a literature review indicates that these methods can effectively address challenges such as noise sensitivity, non-stationarity, and long calibration times, and significantly mitigate these issues through novel classification frameworks and signal processing techniques (Tibermacine et al., 2024). For instance, the STaRNet model integrates multi-scale spatio-temporal convolutional neural networks with riemannian geometry, maps manifold-based features to the tangent space via a matrix logarithm operation for decoding, and achieves an average classification accuracy of 83.29% on the BCI Competition IV 2a dataset without any preprocessing Wang et al. (2024). Similarly, the DFBRTS method employs a dichotomous filter bank combined with riemann tangent space mapping for feature extraction in each sub-band, supplemented by a lightweight CNN for classification, outperforming existing benchmarks in both four-class and two-class tasks Xiong et al. (2025). Meanwhile, the multi-branch Riemannian geometric network framework proposed by Shi et al. (2024), overcomes the training challenges posed by the inability of gradient descent to be directly applied to riemannian manifolds by designing a fusion loss function. Furthermore, the end-to-end EEG-SPDNet proposed by Wilson et al. (2025) demonstrates that deep riemannian networks can learn more complex filtering patterns than traditional bandpass filters and achieve high-performance decoding by leveraging physiologically plausible frequency regions. For zero-shot cross-subject scenario, Manivannan and Lakshminarayan (2025) proposed a geometry-aware deep congruence network that learns discriminative and subject-invariant covariance representations through deep congruence transformations, improving cross-subject accuracy by 3–4%. These studies confirm that manifold-based modeling accurately capture the nonlinear electrode relationships among electrodes in EEG signals while effectively suppressing non-stationarity and inter-subject variability.

However, despite the inherent advantages of riemannian geometry in characterizing the nonlinear structure of EEG signals, existing riemannian methods suffer from two critical limitations. First, core operations such as riemannian mean calculation and tangent space projection lack the ability to actively model noise. The bias of the mean vector of EEG signals to the noisy trials deteriorates the covariance matrix in the manifold space and contaminates the resulting feature representations (Alimardani et al., 2017). Second, by often neglecting variations in data distribution, these methods suffer from significant performance degradation in cross-subject or few-shot settings, where limited data fails to support stable learning of the Riemannian geometric structure (Zhuo et al., 2024).

To overcome the above bottlenecks, we propose novel Riemannian geometry-based deep learning framework for EEG-MI decoding, named Perturbation-aware Differential Riemannian Attention Mechanism (PADA). Unlike existing methods that commonly adopt static covariance descriptors (Wang et al., 2024; Wilson et al., 2025), PADA constructs a dynamic learning process that actively enhances feature robustness and geometric stability through four synergistic components. This module goes beyond simple covariance estimation by incorporating a learnable amplitude amplification factor and an eigenvalue rectification strategy, which rigorously ensures positive definiteness while preserving the inherent local geometric structure of the data.

Our framework’s main novelty lies in the combination of a two-stream differential attention scheme and a tangent-space dynamic perturbation mechanism, while the learnable covariance mapping, BiMap, ReEig, and manifold projection serve as supporting geometric components. We project SPD matrices into the tangent space at the Log-Euclidean mean to reduce the effects of noise and limited samples, then apply controlled perturbations, including scaling and additive noise, within this vectorized space to probe feature instability and improve robustness. The TSDA-Attn module fuses the original amplified manifold features with the perturbed features so that correlations that fluctuate under tangent-space noise receive lower attention. In this design, the perturbed stream acts as a sensitivity indicator, while the direct stream preserves intrinsic manifold structure, and the final representation is mapped back to Euclidean space for classification. As pointed out in (Alimardani et al., 2017; Zhuo et al., 2024), conventional Riemannian methods lack active adaptability to noise and distribution shifts. This study aims to alleviate this limitation, thereby enhancing the model’s cross-scenario generalization performance and robustness.

Our main contributions are summarized as follows:

- We propose a tangent space dynamic perturbation mechanism, which applies geometry-aware scaling and additive noise to SPD features in the Log-Euclidean tangent space, and enhances feature diversity through a curriculum-based annealing strategy. This significantly improves the model’s robustness against the inherent spatiotemporal variability and noise of EEG signals, which is fundamentally different from existing Riemannian methods that rely on static covariance descriptors.
- We design a two-stream manifold differential attention architecture, which is not a simple dual-path parallel structure. Instead, it employs a learnable differential fusion module to perform differentiated comparison between original manifold features and perturbed tangent-space projection features, thereby eliminating noise-sensitive correlations and achieving more robust nonlinear Riemannian representation learning.
- A private EEG dataset was collected from 227 participants performing multi-paradigm tasks, providing an additional benchmark for evaluating the proposed model under limited-sample conditions. The experimental results show that PADA achieves competitive decoding performance and exhibits strong potential for robust BCI applications.

We evaluate the proposed method on two public benchmark datasets, BCI Competition IV-2a and Zhou2016, as well as a private EEG dataset. The results show that PADA achieves competitive clean-condition decoding performance while maintaining stable behavior in challenging scenarios. These findings suggest that PADA is a promising framework for robust MI-EEG decoding in practical BCI applications.

## 2 Proposed method

In this section, we introduce the proposed Riemannian geometry deep learning classifier for EEG-based MI classification, with its overall architecture illustrated in Figure 1. We first extract spatial–temporal features from raw EEG signals using a two-layer convolutional network. These features are mapped onto the Riemannian manifold via an overlapping sliding window and a learnable covariance estimation module. To capture complex nonlinear relationships, we design a Two-stream Differential Manifold Attention (TSDA-Attn) mechanism. This mechanism comprises two streams: one processes features directly on the manifold, while the other injects diversity via a tangent space dynamic perturbation module. Finally, the refined manifold features are selectively mapped back to Euclidean space and classified using a linear layer.

**Figure 1:**
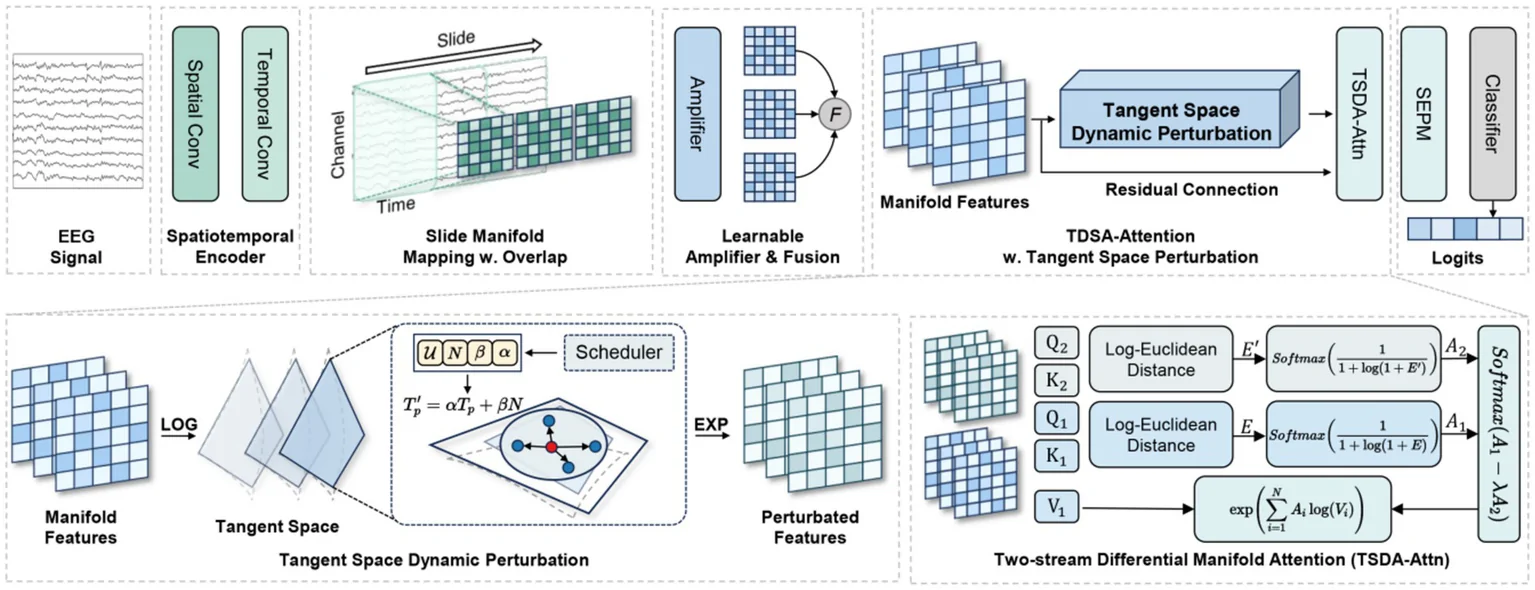
The overall architecture of our proposed method. Spatiotemporal feature extraction followed by transformation to the Riemannian manifold. The tangent space dynamic perturbation module reparameterizes features by injecting noise in the tangent space. The TSDA-Attn mechanism fuses direct manifold attention with perturbed attention.

PADA adopts a hybrid Euclidean-Riemannian architecture for EEG classification. Given a 22-channel, 900-sample input, the network first applies two Euclidean convolutional layers (a 1 × 22 convolution followed by a 1 × 12 convolution) to extract low-level temporal–spatial features. The resulting representation is transformed into SPD matrices by the E2R module partitions the temporal dimension into three overlapping segments (windows) and maps each segment to a 20 × 20 Riemannian representation (with init_scale set to 0.7). The SPD features are then refined by Riemannian batch normalization and alignment, followed by a DiffAttentionManifold block that performs differential attention directly on the manifold. After SPD rectification and manifold dropout (with probability 0.1), the features are projected to the tangent space, yielding a 9 × 19 × 5 representation, which is flattened and fed into a final linear classifier to output the four motor-imagery classes.

### 2.1 Signal mapping

- *Spatiotemporal Encoder*: Given an EEG signal epoch $x\in R^{C\times T}$ , where C denotes the number of channels and T represents the number of time points, we employ a two-layer convolution operation.

The first layer, Conv1, performs spatial filtering with a kernel size of (*C*, 1) (Equation 1):


$$x_{1}={\text{Conv}}_{1}={\text{Conv}}_{C\times 1}(x)$$ (1)

The output is normalized via batch normalization (Equation 2),


$$x_{1}^{'}=\mathrm{BN}(x_{1})\;$$ (2)

The second layer, Conv2, extracts temporal features using a kernel of size (1, *F_t_*), where *F_t_* is the temporal filter length. Padding is applied to preserve the temporal dimension (Equation 3):


$$x_{2}={\text{Conv}}_{2}(x_{1}^{'})={\text{Conv}}_{1\times F_{t}}(x_{1}^{'})$$ (3)

Following a second batch normalization $x_{2}^{'}=\mathrm{BN}(x_{2})$ , we obtain the refined feature representation $\;x_{2}^{\prime }\in R^{C^{\prime }\times T^{\prime }}$ .

- *Slide Manifold Mapping with Overlap:* The features $x_{2}^{\prime }$ are mapped to the Riemannian manifold of SPD matrices using an overlapping sliding window. The temporal dimension is segmented into patches of length *L*= $T^{\prime }/E$ with stride *S* = *max*(1, L(1−R) ), where E is the number of windows and R is the overlap ratio.

For each filtered patch x̂p of size C′ × L, where C′ denotes the number of feature channels and L denotes the temporal length of the window, we apply a learnable covariance estimation procedure. First, we center the data by removing the temporal mean (Equation 4):


$${\tilde{x}}_{p}=x_{p}-\frac{1}{L}\sum_{t=1}^{L}x_{p,c,t}$$ (4)

A learnable filter $W_{f}\in R^{C^{\prime }\times C^{\prime }}$ is applied to the transposed features to enhance interaction: ${\hat{x}}_{p}^{T}={\tilde{x}}_{p}^{T}W_{f}$ . The covariance matrix is then estimated (Equation 5):


$${\mathrm{cov}}_{p}=\frac{1}{L-1}{\hat{X}}_{p}{\hat{X}}_{p}^{T}$$ (5)

To ensure positive definiteness, we apply regularization: ${\mathrm{cov}}_{p}^{\prime }={\mathrm{cov}}_{p}+\mathrm{\epsilon I}$ . Finally, the matrix is projected onto the SPD manifold via a bilinear mapping $W_{\mathrm{bm}}$ and eigenvalue rectification:


$$S_{p}=\text{ReEig}W_{\mathrm{bm}}^{T}{\mathrm{cov}}_{p}^{'}W_{\mathrm{bm}}\;$$ (6)

Where $\text{ReEig}(X)=\text{Udiag}(\max(\lambda_{i},\epsilon ))U^{T}$ . This results in asequence of SPD matrices $S\in R^{B\times P\times C^{\prime }\times C^{\prime }}$ .

### 2.2 Tangent space dynamic perturbation module

Unlike existing methods (e.g., EEG-SPDNet (Wilson et al., 2025), STaRNet (Wang et al., 2024)) that rely on static covariance descriptors and lack active modeling of noise and distribution shifts, we design a Tangent Space Dynamic Perturbation module. Notably, this module is not merely a simple data augmentation strategy; rather, it exploits the local Euclidean property of the manifold tangent space to apply adaptive scaling and additive noise while preserving the global geometric structure of SPD matrices, thereby enhancing the model’s adaptability to EEG signal non-stationarity. This module is not applied globally but is strategically embedded within the second branch of our attention mechanism to specifically enhance feature diversity and improve robustness.

Concretely, given an input SPD matrix $S_{\text{in}}$ , we first compute the Log-Euclidean mean $\bar{S}$ of the batch. The matrix is mapped to the tangent space at $\bar{S}$ (Equation 7):


$$T_{\text{in}}=\log_{\bar{S}}(S_{\text{in}})=\log({\bar{S}}^{-1/2}S_{\text{in}}{\bar{S}}^{-1/2})\;$$ (7)

We then apply a mixed perturbation strategy combining scaling and additive noise (Equation 8):


$$T_{\text{in}}^{'}=\alpha T_{\text{in}}+\mathrm{\beta N}$$ (8)

Where $\alpha \sim U(1-\frac{\eta }{2},1+\frac{\eta }{2})\;$ is a scaling factor, *N* is a standard normal random matrix, and *β* is a noise level parameter. The perturbed tangent matrix is mapped back to the SPD manifold at the mean point:


$$S_{\text{pert}}=S^{1/2}\exp(T_{\text{in}}^{\prime })S^{1/2}\;$$ (9)

The noise parameter *β* is annealed during training: $\beta_{t}=\beta_{0}\max(\mathrm{tol},1-\frac{t}{T_{\max}})\;$ .

### 2.3 Two-stream differential manifold attention (TSDA-Attn)

To capture nonlinear relationships and mitigate noise, we propose the TSDA-Attn mechanism. This is different from the existing methods that only perform single-flow self-attention calculations on the original SPD manifold and lack the ability to identify and suppress noise-sensitive connections. It consists of a learnable amplifier and two parallel attention streams that are differentially fused.

- *Learnable Amplifier & Fusion*: First, the input sequence S is modulated to emphasize informative features (Equation 10):

$$S_{\mathrm{amp}}=\exp(s)\cdot S$$ (10)

Where *s* is a learnable scalar,exp.(s) provides a positive amplification factor.

- *Stream 1: Direct Manifold Attention*: This stream captures intrinsic geometric structures from the amplified features $S_{\mathrm{amp}}$ . We generate query (*Q*_1_) and key (*K*_1_) representations via learnable SPD transformations *W*_*Q*1_, *W*_*K*1_ (Equation 11):

$$Q_{1}=W_{Q1}^{T}S_{\mathrm{amp}}W_{Q1},\;\;\;\;K_{1}=W_{K1}^{T}S_{\mathrm{amp}}W_{K1}\;$$ (11)

The attention energy is computed using the Log-Euclidean distance (Equation 12):


$$E_{\mathrm{ij}}=\|\log(Q_{1,i})-\log(K_{1,j})\|F^{2}$$ (12)

The attention map *A*_1_ is obtained via softmax (Equation 13):


$$A_{1}=\text{softmax}(\frac{1}{1+\log(1+E)})\;\;$$ (13)

- *Stream 2*: Perturbed Tangent Attention: In parallel, this stream injects perturbations to improve generalization. The amplified features Samp are passed through the Tangent Space Dynamic Perturbation Module defined previously to obtain Spert. We then compute Q_2_ and K_2_ from Spert and calculate the secondary attention map A_2_ similarly to Stream 1 (Equation 14):

$$A_{2}=\text{softmax}(\frac{1}{1+\log(1+E^{\prime })})\;$$ (14)

Where *E′* is the distance matrix derived from the perturbed features.

- Differential Fusion and Aggregation: The two attention maps are fused using a learnable differential weighting scheme. Learnable vectors $\lambda_{q1}$ , $\lambda_{k1}$ , $\lambda_{q2}$ , and $\lambda_{k2}$ are introduced to adaptively balance the contributions of the two attention maps. In our implementation, these parameters are $d_{\lambda }$ -dimensional vectors, where $d_{\lambda }$ equals the output SPD dimension of the attention module. The initialization term $\lambda_{\text{offset}}$ is a scalar (Equation 15).

$$\lambda =\exp(\sum_{r=1}^{d_{\lambda }}\lambda_{q1,r}\lambda_{k1,r})-\exp(\sum_{r=1}^{d_{\lambda }}\lambda_{q2,r}\lambda_{k2,r})+\lambda_{\text{offset}}$$ (15)

Where $\lambda_{q1}$ , $\lambda_{k1}$ , $\lambda_{q2}$ , and $\lambda_{k2}$ ∈ $R^{d_{\lambda }}$ are learnable vectors, *d_λ_* denotes the vector dimension and is set equal to the output SPD dimension dout, and *λ*offset is a scalar initialization term. The summation denotes a dot product over the *d_λ_* dimension.

The final attention map is $A=\text{Softmax}(A_{1}-\lambda A_{2}\;)$ . By subtracting the attention map derived from perturbed features, the model performs a soft-thresholding operation, explicitly down-weighting noise-sensitive connections to enhance the robustness of the final representation. This is an essential improvement to the existing attention paradigm.

We aggregate the value matrices $V=W_{V}^{T}S_{\mathrm{amp}}W_{V}$ using the Log-Euclidean mean weighted by A (Equation 16):


$$O=\exp(\sum_{i=1}^{P}A_{i}\log(V_{i}))\;$$ (16)

### 2.4 Classifier

- *Selective Euclidean Space Mapping* (*SEPM*): To transition from the Riemannian manifold to Euclidean space for classification, we project the aggregated SPD feature *O* to the tangent space (Equation 17). To further improve generalization, we apply a random masking mechanism directly in the tangent space (Equation 18):

$$O_{\tan}=\log(O)$$ (17)

$$O_{\mathrm{euc}}=O_{\tan}⊙\text{Mask},\;\;\text{Mask}∼\text{Bernoulli}(1-p)$$ (18)

Where p is the dropout probability. Unlike previous steps, we do not map Oeuc back to the manifold; instead, it serves as the final Euclidean feature representation.

- *Linear Layer Classifier*: The masked Euclidean features $O_{\mathrm{euc}}$ are flattened into a vector *f* and fed into a linear classifier to compute class scores $s_{c}=W_{c}^{T}f+b_{c}$ . The final probabilities are obtained via softmax (Equation 19):

$$P(y=c|x)=\frac{\exp(s_{c})}{\sum_{c\prime =1}^{C}\exp(s_{c\prime })}$$ (19)

Where C denotes the total number of classes, c is the target class index, and $c^{\prime }$ is the summation index over all classes.

### 2.5 Theoretical insight and intuitive analysis

Having detailed the architectural designs of our proposed modules in Sections A–D, we now provide theoretical and intuitive explanations for why these modules improve robustness and preserve geometric properties.

#### 2.5.1 Regarding geometric property preservation

All our manifold operations are grounded in rigorous Riemannian geometry theory. The tangent space dynamic perturbation module operates in the Log-Euclidean tangent space, which is a local Euclidean approximation of the manifold at the mean point. By mapping the perturbed tangent vectors back to the SPD manifold via exponential mapping (Equation 9), we guarantee that the output matrices remain on the SPD manifold. Specifically, for any symmetric matrix *T* and exp.(*T*) is always positive definite, ensuring the positive definiteness of *Sout*​. Meanwhile, the ReEig regularization (Equation 6) further enforces positive definiteness through eigenvalue thresholding via *ReEig*(*X*) = *U*diag(max(*λi*,*ϵ*))*UT*. This design ensures that perturbations do not compromise the global geometric structure of the manifold.

#### 2.5.2 Regarding the robustness enhancement mechanism

The two-stream differential attention is motivated by a key insight: if a certain feature connection is highly sensitive to small perturbations in the tangent space, then it is also often fragile and unreliable under changes in the true data distribution. Through learnable differential fusion (Equation 15), we force the model to focus on those connections that remain stable under perturbation, which correspond to the truly discriminative neural activity patterns in the EEG signal, rather than the false correlations caused by noise. This mechanism is essentially an intrinsic regularization that enables self-supervised robust feature selection without the need for external noise labels.

#### 2.5.3 Regarding the curriculum-based annealing strategy

The perturbation strength *βt* = *β*0max(tol,1 − *t*/*T*max) decays during training, which aligns with the curriculum learning framework. In early training stages, larger perturbations force the model to learn broad, invariant representations; in later stages, smaller perturbations allow fine-tuning to the local structure of the true data distribution. It has been proven effective in improving generalization across various deep learning tasks.

## 3 Experimental analysis

### 3.1 Datasets description

We evaluate the proposed PADA framework on two public benchmark datasets: BCI Competition IV 2a (Tangermann et al., 2012), Zhou2016 (Zhou et al., 2016) and CEP-MI dataset. A summary of the dataset statistics is provided in Table 1.

- *BCI Competition IV* 2*a*: This dataset comprises EEG recordings from nine participants (A01-A09) engaged in a cue-based BCI paradigm. The paradigm involves four motor imagery tasks: left hand, right hand, both feet, and tongue. Each participant completed two sessions on separate days, with each session consisting of six runs. Each run contains 48 trials (12 per class), resulting in a total of 288 trials per session. The signals were sampled at 250 Hz and bandpass-filtered between 0.5 and 100 Hz with a 50 Hz notch filter.
- *Zhou*2016: This dataset contains EEG data from four subjects (S1-S4) performing three types of motor imagery tasks: left hand, right hand, and foot. Each subject participated in 3 sessions, with intervals ranging from days to months. Each run consists of 75 trials (25 per class), sampled at 250 Hz.
- *Cue-Execution dual-phase MI paradigm* (*CEP-MI*): We employed a novel Cue-Execution dual-phase MI paradigm consisting of two blocks, as illustrated in Figure 2A. To facilitate comparison with existing literature, Block 1 followed the classic arrow paradigm. In this block, images depicting left hand, right hand, left foot, right foot were sequentially presented at the center of the screen. Subject were instructed to perform the cued motor imagery for 4 s according to the displayed image. Each trial was separated by a 1-s inter-trial interval, during which a fixation cross was presented on the screen. Each movement type comprised 12 trials, resulting in a total of 96 trials. Figure 2B illustrated the timeline of the cue-execution MI task. In each trial, a fixation cross appeared on a black screen for 1 s. Then, a countdown arrow (3, 2, 1) was shown. During the countdown, participants performed motor imagery. After the countdown, they made a slight limb movement together with MI, followed by a 1 s rest.

**Table 1:** Summary of the public dataset and laboratory dataset used in the experiments.

Datasets	Channels	Subject	Classes	Fs (Hz)	Train trials
Zhou2016 (Zhou et al., 2016)	3	4	3 (Left hand, right hand, both feet)	250	150 (75 per run)
BCI_IV-2a (Tangermann et al., 2012)	22	9	4 (Left hand, right hand, both feet, tongue)	250	288 (48 per run)
CEP-MI dataset	60	227	4 (Left hand, right hand, left foot, right foot)	250	24 (run 1) 34 (run 2)

**Figure 2:**
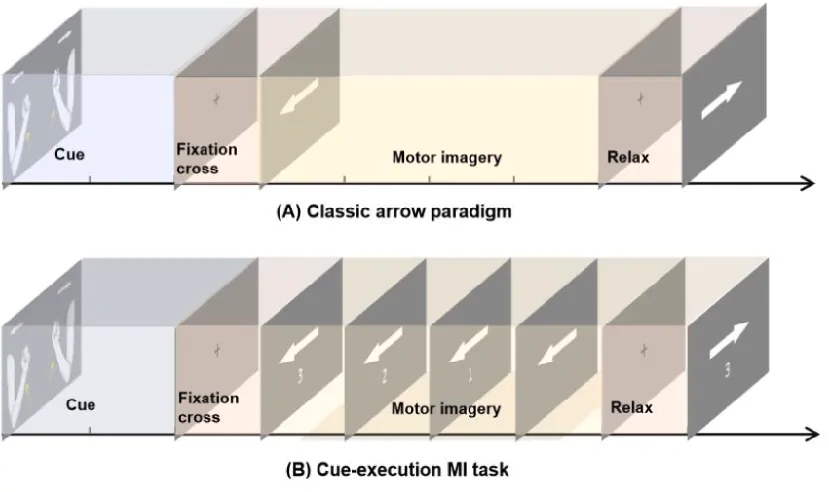
Experimental procedures of the motor imagery tasks **(A)** Classic arrow paradigm with a 4-s MI period **(B)** Cue-execution task with countdown arrows before subtle movement execution.

The CEP-MI dataset comprised 227 healthy subjects (10 left-handed and 217 right-handed; 167 males and 60 females), and is referred to as the CEP-MI in this work. The EEG data utilized in this study were collected at Inner Mongolia University. All participants were recruited from the university campus. The experiment was conducted in accordance with the Declaration of Helsinki, and the protocol was approved by the Ethics Committee of Inner Mongolia University. Prior to the experiment, each subject received a thorough explanation of the procedure and provided written informed consent. Exclusion criteria included a history of neurological or musculoskeletal disorders.

### 3.2 Data preprocessing

EEG activity was recorded by Neuroscan system using the 69 Ag/AgCl electrodes placed according to the international 10/20 system in a quiet room. The impedance of each electrode was kept below 10 kΩ and were made against FCz as reference. Offline pre-processing of EEG data was performed with MNE software. The signal was filtered with band-pass filter (0.5–45 Hz) and eye movement artifacts were removed by using independent component analysis. The oscillatory features were obtained by direct band-pass filtering of the EEG signals, rather than by spectral estimation. Specifically, we used a 5th-order Butterworth band-pass filter with zero-phase forward-backward filtering. The canonical sub-bands were defined as alpha (8–13 Hz), beta (13–30 Hz), theta (4–8 Hz), and gamma (30–47 Hz). The sampling rate was downsampled to 250 Hz (Li et al., 2021). After excluding non-scalp electrodes, EEG data from a total of 60 EEG electrodes were retained.

To eliminate inter-subject and inter-channel variability, Z-score normalization was applied independently to each subject and each channel. For a given subject, the mean and standard deviation were calculated exclusively from the training set. These statistics were then used to normalize both the training set and the corresponding test set for that subject. This subject-wise, training-set-dependent strategy ensures that the test data do not contribute to the estimation of normalization parameters, thereby preventing any information leakage and ensuring unbiased performance evaluation. The normalization is defined as (Equation 20):


$$x_{\mathrm{norm}}=\frac{x-\mu }{\sigma }$$ (20)

Where *x* denotes the original EEG signal, while *μ* and σ represent the mean and standard deviation across the time dimension, respectively. This step preserves temporal dynamics while mitigating the adverse effects of amplitude variability.

### 3.3 Implementation details

All models and baselines were trained and evaluated on a single computer equipped with a 14th Gen Intel(R) Core(TM) i9-14900HX CPU and an NVIDIA GeForce RTX 4090 GPU (8 GB memory).

For training configuration, a cosine annealing scheduler (T₀ = 30) was employed to dynamically adjust the learning rate. The batch size was flexibly configured with values of 16, 32, or 256 depending on the dataset. A general dropout rate of 0.5 was applied to prevent overfitting. Weight decay of 0.05 was used as the primary setting, though a smaller value of 0.0001 was also explored in specific experiments to evaluate its regularization effect. Additionally, the model included specific hyperparameters such as init_scale for initialization and types of feature perturbation.

For the BCI2a dataset, hyperparameters were fixed based on a small ablation study rather than an exhaustive search. The model was trained with AdamW using a learning rate of 6e-4 and weight decay of 0.05, with label smoothing set to 0.1 and λ₁ = λ₂ = 0. Training ran for 500 epochs without early stopping. Manifold dropout with probability 0.1 was applied in the final pipeline.

### 3.4 Evaluation protocol

This study adopts a subject-dependent evaluation paradigm, where models are individually trained and tested for each subject. We employ a random split strategy with a 1:1 ratio to divide each subject’s data into training and test sets. No separate validation set is held out; instead, a portion of the training set is reserved for early stopping and hyper parameter tuning. To rigorously assess generalization on unseen subjects, we supplement evaluation on the BCI IV-2a dataset with a leave-one-subject-out (LOSO) cross-validation experiment, where data from one subject is sequentially used as the test set and the remaining eight subjects as the training set, resulting in nine folds. Importantly, across all experiments, we strictly prevent data leakage during preprocessing: all statistical parameters (mean and standard deviation) for Z-score normalization are computed exclusively from the training set and then applied to the test set, ensuring that test data does not participate in any training process, thus guaranteeing the reliability and reproducibility of our evaluation results.

Model performance was evaluated using both accuracy and the estimated Kappa score, where the estimated Kappa serves as a chance-corrected accuracy measure. Subject-wise test accuracies were analyzed using the Wilcoxon signed-rank test. To address the issue of multiple comparisons, we applied Holm correction to adjust the *p*-values. In addition to *p*-values, we report the 95% confidence intervals of the paired accuracy differences to quantify estimation uncertainty, and we report the rank-biserial correlation as the effect size to reflect the practical magnitude of the observed differences.

## 4 Experimental results

### 4.1 Comparison with state-of-the-art

Table 2 reports the clean-condition classification accuracy on the BCI Competition IV-2a dataset. PADA achieves an average accuracy of 81.44%, showing strong competitiveness among the compared methods. In particular, PADA obtains the best or tied-best performance on A01, A07, and A09, and remains close to the leading results on A02 and A03. These results indicate that PADA can maintain reliable clean-condition decoding performance, which provides a solid basis for further evaluating its robustness under noisy conditions.

**Table 2:** Classification accuracy (%) on the BCI Competition IV 2A dataset.

Model	A01	A02	A03	A04	A05	A06	A07	A08	A09	Avg	K_est_
SSCSP (Arvaneh et al., 2011)	76.74	58.68	81.25	57.64	38.54	48.26	76.39	79.17	78.82	66.17 ± 14.85	0.549
FBCSP (Ang et al., 2008)	77.78	55.56	79.51	63.19	53.47	46.88	86.81	81.25	68.06	68.06 ± 13.30	0.574
ShallowConvNet (Schirrmeister et al., 2017)	77.08	51.04	86.81	66.32	69.10	52.43	81.25	86.11	79.80	72.22 ± 12.71	0.630
EEGNet (Lawhern et al., 2018)	89.38	60.42	92.36	66.32	70.14	59.72	86.46	80.10	83.33	76.10 ± 11.83	0.681
DRDA (Zhao et al., 2020)	83.19	55.14	87.43	75.28	62.29	57.15	86.18	83.61	82.00	74.70 ± 12.22	0.663
DRDW (She et al., 2023)	83.29	63.97	90.30	76.94	69.34	60.08	89.31	82.35	82.81	77.60 ± 10.23	0.701
EEGConformer (Song et al., 2022)	89.23	58.33	**92.66**	65.28	49.31	59.72	93.40	81.25	80.21	74.34 ± 15.57	0.658
MI-CAT (Zhang et al., 2023)	**90.62**	54.51	91.32	72.57	63.19	62.85	87.15	85.07	84.03	76.74 ± 13.01	0.690
EEG-TransNet (Ma et al., 2024)	86.46	48.26	87.15	69.79	52.78	57.29	86.81	80.56	84.38	72.61 ± 15.04	0.635
MAtt (Pan et al., 2022)	88.54	60.42	82.99	73.61	56.25	56.60	88.89	82.29	83.33	74.77 ± 12.77	0.664
TCNet (Han et al., 2024)	84.03	64.93	92.01	66.32	73.61	61.81	87.15	82.64	81.94	77.16 ± 10.20	0.696
ATCNet (Altaheri et al., 2022)	81.57	**67.15**	83.46	**90.97**	**93.03**	**83.85**	91.88	**90.99**	84.83	85.27 ± 7.58	0.804
SecNet (Liang et al., 2025)	—	—	—	—	—	—	—	—	—	87.08	
PADA (Ours)	**90.62**	66.67	91.67	76.39	78.12	61.11	**94.44**	88.19	**85.76**	81.44 ± 11.01	0.752

The best results are highlighted in bold.

We further evaluated the statistical significance of the accuracy differences between PADA and each baseline under the LOSO protocol using the paired Wilcoxon signed-rank test, with Holm–Bonferroni correction applied for multiple comparisons. The results are summarized in Supplementary Table S1. PADA achieves statistically significant improvements over most competing methods under the subject-wise evaluation protocol. In particular, the gains over classical CSP-based methods and several deep learning baselines remain significant after Holm-Bonferroni correction, suggesting that PADA provides a robust advantage across subjects. By contrast, the improvements over TCNet and MI-CAT are positive but do not survive multiple-comparison correction, which implies that these recent models are closer competitors. ATCNet is the only baseline with a higher mean accuracy than PADA, but the paired difference is not statistically significant, indicating that PADA remains competitive but is not uniformly superior to every strong recent method. See Appendix.4 Statistic analysis for details.

As shown in Table 3, PADA demonstrates exceptional performance on the Zhou2016 dataset with an average accuracy of 92.79%, surpassing the second-best methods (Conformer and MI-CAT) by 1.61%. Notably, our approach consistently attains the highest accuracy across all four subjects. For subject S4, PADA achieves 94.25%, exceeding the nearest competitor by over 3%. The standard deviation of 1.47% across subjects is significantly lower than that of other methods, underscoring the robustness of our framework.

**Table 3:** Classification accuracy (%) on the zhou2016 dataset.

Model	S1	S2	S3	S4	Avg.
EEGNet (Lawhern et al., 2018)	84.38	75.86	82.42	80.89	80.89 ± 3.16
EEGConformer (Song et al., 2022)	**92.71**	88.51	92.31	91.18	91.18 ± 1.64
MI-CAT (Zhang et al., 2023)	**92.71**	88.51	92.31	91.18	91.18 ± 1.64
EEG-TransNet (Ma et al., 2024)	90.61	80.46	91.21	87.43	87.43 ± 4.27
MAtt (Pan et al., 2022)	87.43	83.91	90.11	86.21	86.92 ± 2.24
PADA (Ours)	**92.71**	**90.80**	**93.41**	**94.25**	**92.79 ± 1.27**

The best results are highlighted in bold.

To evaluate the model’s discriminability under different duration and limb conditions, we conducted four independent binary classification tasks on the CEP-MI dataset, each corresponding to a specific combination of limb type (hand vs. feet) and MI duration (classic MI vs. CEP MI). The results are summarized in Table 4. As shown in the table, PADA significantly outperforms ATCNet, which had previously achieved the best results on public datasets, achieving an improvement of approximately 9.4 to 12%. At the same time, it achieved accuracy improvements of over 6 and 13% compared to EEGNet and FBCSP, respectively. PADA maintains robust performance even with sparse data, highlighting its suitability for real-world scenarios with limited samples. Furthermore, it is worth noting that ATCNet performs worse than EEGNet and FBCSP, possibly because it is unable to learn discriminative features under the constraints of small-sample data.

**Table 4:** Binary classification accuracy (%) for each independent motor imagery task on cep-mi dataset.

Model	Classic MI task (left hand *vs.* right hand)	CEP MI task (left hand *vs.* right hand)	CEP MI task (left feet vs. right feet)	Classic MI task (left feet vs. right feet)
FBCSP (Ang et al., 2008)	57.05	61.33	54.56	54.19
EEGNet (Lawhern et al., 2018)	64.42	65.68	61.51	66.39
ATCNet (Altaheri et al., 2022)	61.04	63.75	61.71	63.29
PADA (Ours)	70.46	71.01	65.78	65.13

Each column represents an independent binary classification task [limb type (hand vs. feet) × MI task (classic MI vs. CEP MI)]. The dataset contains four MI classes in total (Table 1), while this table reports per-task discriminability rather than a four-class multiclass accuracy.

To address the concern regarding cross-subject generalization, an additional leave-one-subject-out (LOSO) experiment were conducted on the BCI Competition IV-2a dataset. Under the LOSO protocol, the proposed PADA model achieves an accuracy of 39.54% ± 9.62% (see Supplementary Table S2), which is substantially lower than its subject-dependent performance.

### 4.2 Robustness analysis

To evaluate the robustness of the proposed method against low SNR, we test the trained models on data injected with varying levels of Gaussian noise. Five tests were conducted for each noise level, and the average was taken as a measure of the model’s robustness (Equation 21).


$$X_{\text{noisy}}=X+N(0,{\left(\alpha \cdot \sigma_{X}\right)}^{2})$$ (21)

Table 5 and Table 6 illustrate the performance degradation under noise for BCI Competition IV 2a and Zhou2016, respectively. Table 5 shows that PADA maintains high accuracy on BCI Competition IV 2a even under severe noise conditions. At a 15% noise level, it outperforms several baselines evaluated on clean data. Even at 30% noise, our model achieves accuracy comparable to the Riemannian geometry classifier MAtt on clean data. Similar trends are observed on the Zhou2016 dataset in Table 6, confirming the efficacy of the TSDA-Attn mechanism in suppressing noise.

**Table 5:** Robustness analysis on the BCI Competition IV 2a under varying noise levels.

Subject	5%	10%	15%	20%	25%	30%
A1	90.28	89.93	87.50	87.50	88.19	86.46
A2	63.54	64.58	62.85	62.50	61.11	56.94
A3	89.93	88.19	89.58	89.58	84.38	85.76
A4	73.61	74.31	72.57	71.53	70.83	69.79
A5	74.31	71.88	70.14	68.40	68.40	63.54
A6	61.11	59.03	56.60	56.60	55.56	53.82
A7	94.10	93.75	93.40	92.01	91.67	89.93
A8	87.50	86.81	84.03	84.38	80.90	82.64
A9	84.03	85.07	84.38	82.29	82.29	81.94
Avg.	**79.82 ± 11.43**	**79.28 ± 11.54**	**77.89 ± 12.10**	**77.20 ± 12.05**	**75.93 ± 11.79**	**74.54 ± 12.94**

The best results are highlighted in bold.

**Table 6:** Robustness analysis on the zhou2016 dataset under varying noise levels.

Subject	0.05	0.10	0.15	0.20	0.25	0.30
S1	91.67	90.63	89.58	86.46	85.42	86.4
S2	90.80	89.66	90.80	88.51	87.36	82.76
S3	91.21	91.21	90.11	87.91	87.91	82.42
S4	90.80	90.80	88.51	87.36	87.36	89.66
Avg.	**91.12 ± 0.36**	**90.57 ± 0.57**	**89.75 ± 0.84**	**87.56 ± 0.75**	**87.01 ± 0.95**	**85.32 ± 2.96**

The best results are highlighted in bold.

To further evaluate the robustness of the proposed PADA method in noisy environments, we compared its performance against several baseline models, including ShallowConvNet, EEGNet, TCNet, and the state-of-the-art SceNet. As shown in Table 7, at a 20% noise level, the classification accuracy of PADA decreased by only 2.78%. Under 50% noise, the accuracy of PADA dropped by only 12%, achieving the best classification performance among the compared models, whereas the SecNet model experienced a drop of 20.4%. These results demonstrate the excellent anti-noise capability of PADA.

**Table 7:** Classification accuracy (%) of various models across noise ratios from 0 to 50%.

Model	0%	10%	20%	30%	40%	50%
ShallowConvNet (Schirrmeister et al., 2017)	72.22	69.21	58.99	49.23	39.00	31.71
EEGNet (Lawhern et al., 2018)	76.10	73.61	63.31	52.64	42.71	37.46
TCNet (Han et al., 2024)	77.16	76.39	67.40	57.72	50.46	43.06
SceNet (Liang et al., 2025)	87.65	86.15	82.60	77.35	70.64	66.93
PADA (Ours)	81.44	80.47	78.66	75.27	71.72	69.02

Figure 3 displays violin plots of the accuracy metric under different noise levels (0 to 50%). As the noise ratio increases, both the mean and the overall distribution of the accuracy metric exhibit a declining trend, with the data distribution progressively shifting toward lower values. This indicates that higher noise levels lead to a reduction in the accuracy metric.

**Figure 3:**
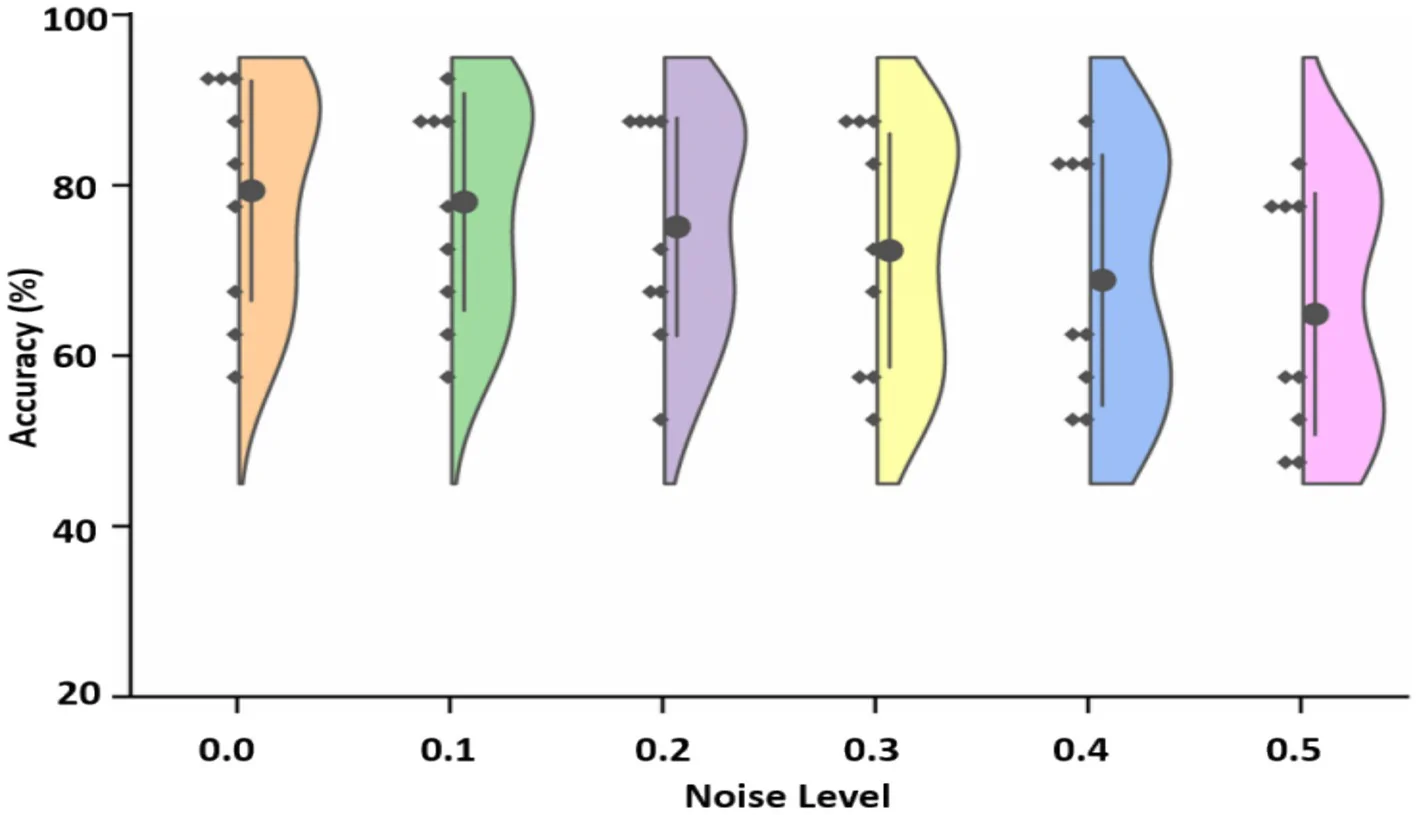
The accuracy distribution of each subject in the PADA under different noise levels. Each colored violin shape represents the data distribution under its corresponding noise condition, with its width indicating data density within value ranges.

In order to study the performance of PADA under actual degraded recording conditions, we conducted three complementary robustness experiments, covering issues such as channel sparsity, channel loss/misalignment, and physiological interference. Detailed results can be found in Appendix.3.

### 4.3 Frequency-specific contributions to MI discrimination

The inhibition of oscillatory activities in the alpha (8–13 Hz) and beta (13–30 Hz) frequency bands within the contralateral sensorimotor region during motor execution and motor imagery tasks has been widely discussed (Srinivasan et al., 2025). The study further compared the classification performance of PADA under three spectral settings: alpha band, beta band, and the whole band. As illustrated in Figure 4, PADA utilizing the whole band achieved statistically significant improvements over single-band inputs in precision for the tongue and right-hand tasks, as well as in recall for the both feet task. In addition, the proposed method utilizing the whole band achieved statistically significant improvements over single-band inputs in precision for the Tongue (*p* < 0.01) and Right-Hand (*p* < 0.05) tasks, as well as in recall for the Feet task (*p* < 0.05), with detailed *p*-values reported in the caption of Supplementary Table S4 and Supplementary Table S5. This stability validates the effectiveness of mapping EEG signals to the Riemannian manifold via block covariance matrices, effectively constructing a feature space resistant to signal fluctuations.

**Figure 4:**
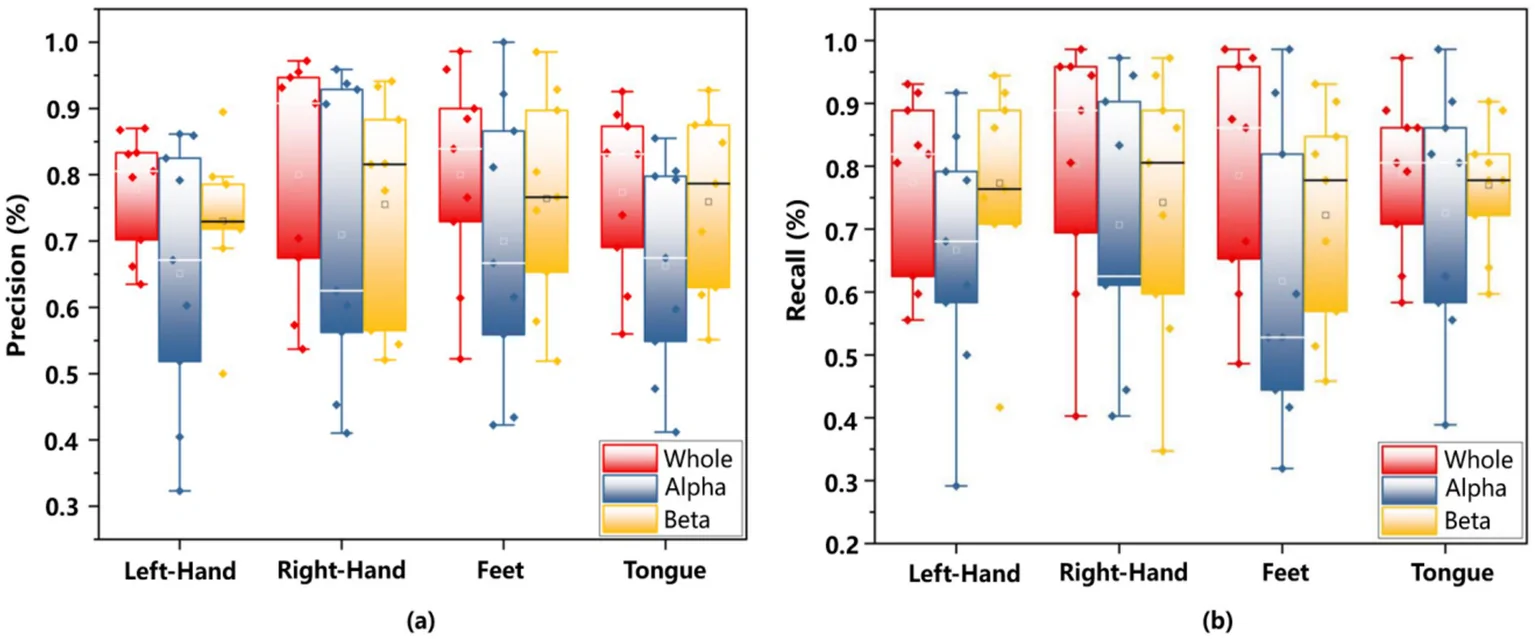
Distribution of precision **(a)** and recall **(b)** scores for the four MI tasks using alpha, beta, and whole band inputs. Box edges represent the interquartile range (25th–75th percentiles), central marks indicate the median, and diamonds denote individual subject scores.

### 4.4 Feature-based visualization

To enhance the interpretability of the model’s decisions and investigate the neural mechanisms underlying the distinction between correct and incorrect classifications, the following analyses were conducted. For each subject, test-set data along with the corresponding model-predicted labels were loaded. Cz-channel data were then segregated into correct and incorrect trials based on prediction accuracy. Following predefined frequency band segmentation (delta, theta, alpha, beta, and gamma), time-frequency power variations within the 0–1.5 s epoch were computed for each trial using Morlet wavelet transforms, generating event-related spectral perturbation (ERSP) plots. Concurrently, the average event-related potential (ERP) waveform across all Cz-channel trials was derived, smoothed via Savitzky–Golay filtering, and normalized. The resulting ERP trajectory was vertically scaled to align with the ERSP frequency range and overlaid onto the spectrogram.

The ERSP and ERP corresponding to trials in which the model made errors versus those it classified correctly are presented in Figure 5. As shown in figure, subjects with high classification accuracy (e.g., A01, A03, A07, A09) exhibited distinct differentiation in ERSP and ERP features between correct and error trials. Correct trials typically showed more significant power changes in specific frequency bands (e.g., theta, alpha, and beta) and sharper ERP waveform peaks, whereas error trials displayed weaker features. In contrast, subjects with low classification accuracy (e.g., A02, A04, A06) demonstrated minimal differences in neural responses between correct and error trials, with substantial overlap in both time-frequency characteristics and ERP waveforms, thereby making it difficult for the model to distinguish between them.

**Figure 5:**
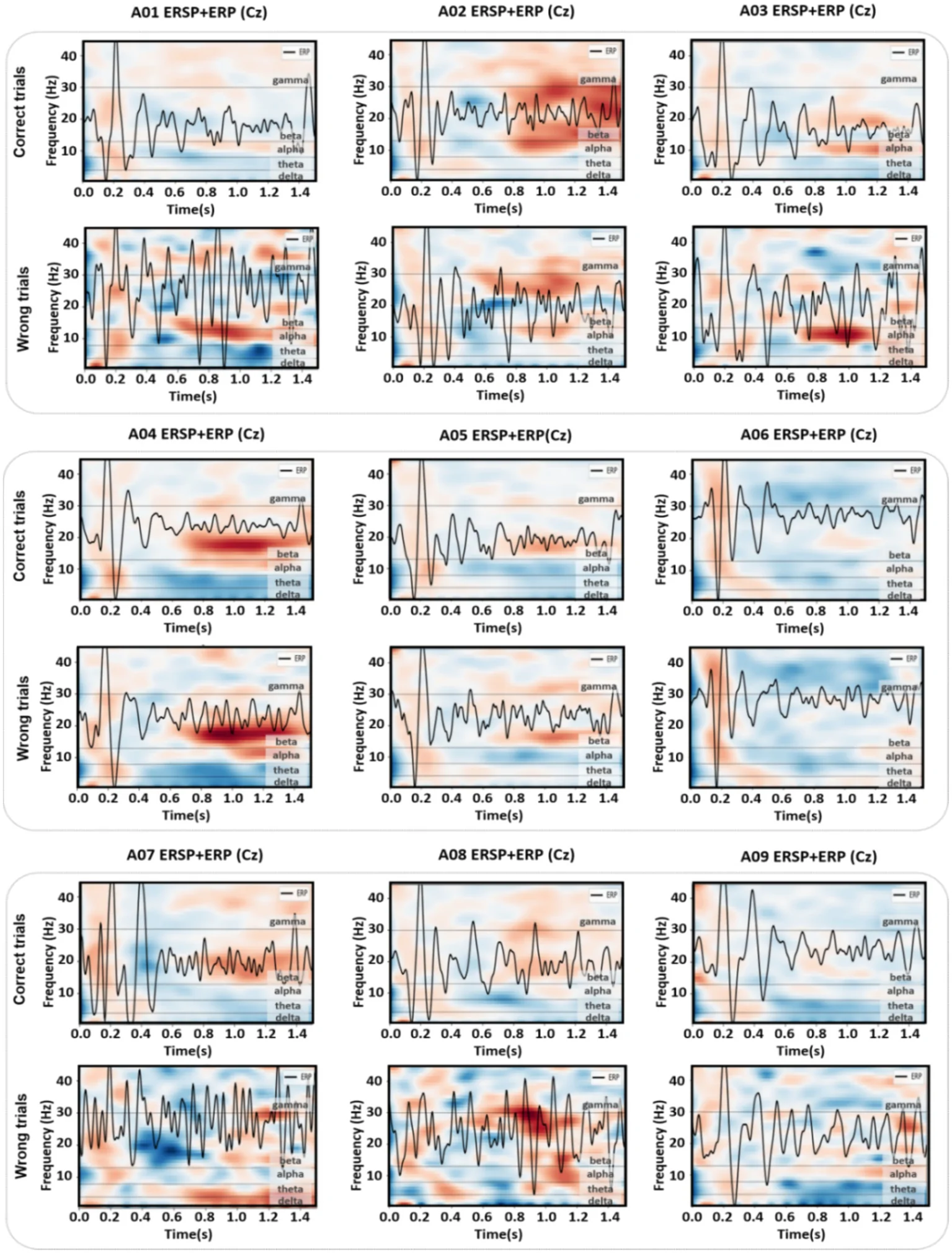
ERSP and ERP results for correctly and incorrectly classified trials. Background colors denote ERSP changes across time and frequency, with red indicating power increase and blue indicating power decrease, while the black line represents the ERP waveform. The horizontal axis indicates time (s), and the vertical axis indicates frequency (Hz).

In addition, we employed t-distributed stochastic neighbor embedding (t-SNE) to visualize discriminative feature learning and validate model efficacy. Distinct four-class clusters (left/right hand, foot, tongue) emerged in 5/9 subjects (A01, A03, A07, A08, A09), confirming effective feature discrimination. Partial left–right hand overlap occurred in S4-S5, though foot/tongue clusters remained separable, as shown in Figure 6. Despite complex distributions in S2/S6, discernible category boundaries persisted, indicating robust feature encoding under challenging conditions. These well-defined clusters collectively demonstrate the model’s capability to extract task-discriminative representations, visually corroborating quantitative classification results (81.44% accuracy).

**Figure 6:**
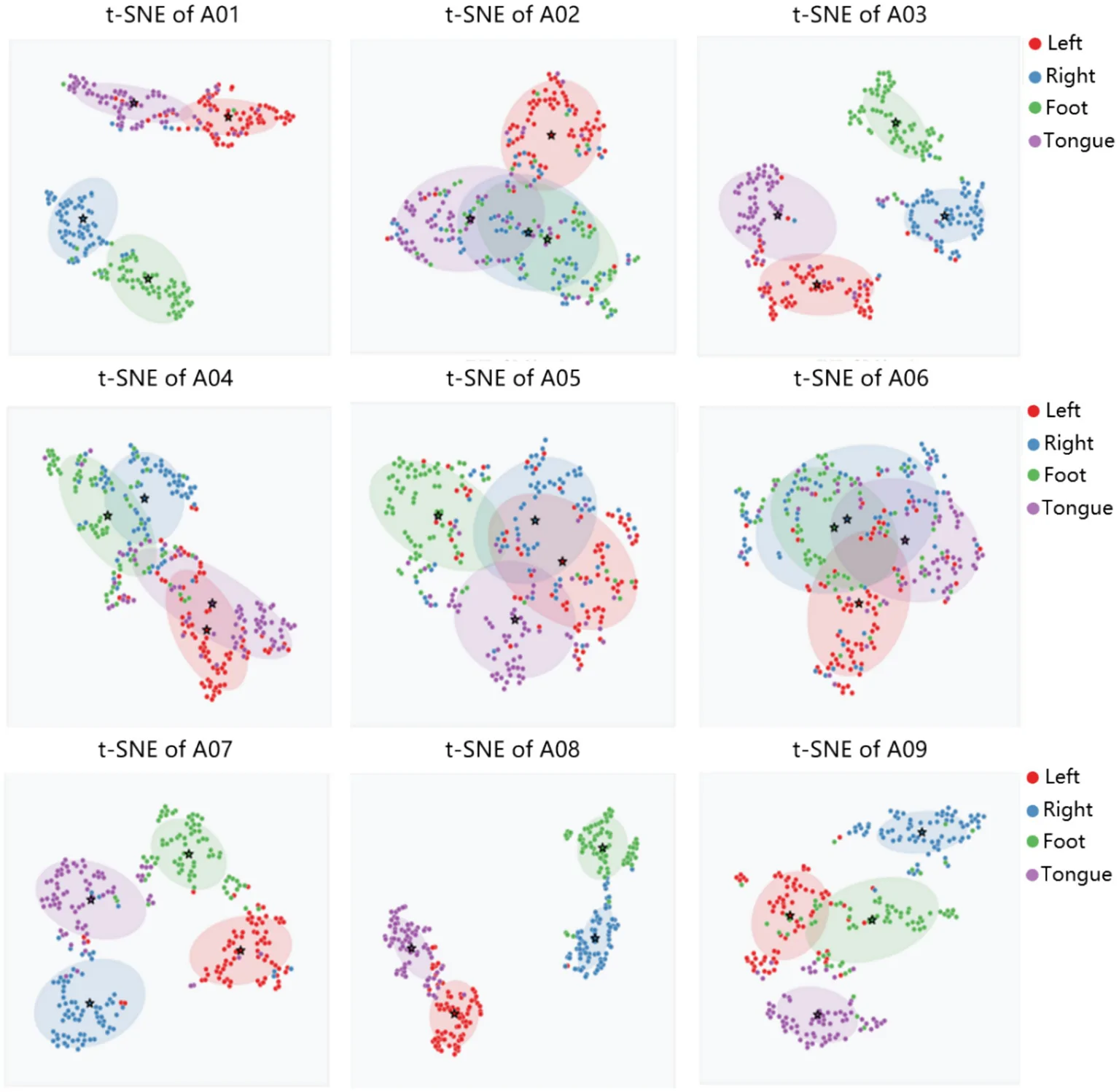
*t*-SNE visualizations of the learned feature space on BCI4-2a. Each panel corresponds to one subject, and colors denote the four MI classes.

### 4.5 Ablation study

To validate the contribution of each component in PADA, we conduct an ablation study on the BCI Competition IV 2a dataset. The results are summarized in Table 8. For more detailed information, please refer to Supplementary Table S4.

- *Impact of the learnable amplifier*: The baseline model achieves 76.31%. Incorporating the learnable amplifier improves performance, with a ratio of 0.7 yielding the best result (76.74%). This suggests that appropriate scaling of SPD features enhances nonlinear representation without compromising geometric stability.
- *Impact of two-stream differential manifold attention* (*TSDA-Attn*): We first evaluate the attention mechanism with only the direct stream (TSDA-Attn (First-stream)). Without the amplifier, it reaches 77.08%; combined with the amplifier (0.7), it improves to 78.16%. This confirms that attention on the manifold effectively captures intrinsic patterns. Subsequently, enabling the second stream with Tangent Space Dynamic Perturbation (TSDA-Attn (Second-stream)) further increases accuracy to 79.03%. This significant gain validates our hypothesis that injecting diversity via tangent space perturbation enhances robustness against spatiotemporal variability.
- *Impact of selective euclidean space mapping* (*SEPM*): Finally, applying the selective masking mechanism in the tangent space prior to classification results in the peak accuracy of 80.86%. This module acts as a regularizer, preventing overfitting by randomly dropping features before the linear classifier.

**Table 8:** Ablation study on BCI competition IV 2a (%).

Configuration	Amp. ratio	First-stream	Second-stream	SEPM	Avg	Gain
Baseline	—	—	—	—	76.31	—
+Amplifier	0.3	—	—	—	76.65	+0.34
+Amplifier	1.2	—	—	—	76.31	+0
+Amplifier	0.7	—	—	—	76.74	+0.43
—	—	√	—	—	77.08	+0.77
+Stream 1	0.7	√	—	—	78.16	+1.85
+Stream 2	0.7	√	√	—	79.03	+2.72
+SEPM (full PADA)	0.7	√	√	√	81.44	+5.13

We investigate the impact of the learnable amplifier (amp.), the two streams of the two-stream differential manifold attention (TSDA-Attn), and the selective Euclidean space mapping (SEPM).

### 4.6 Efficiency analysis

Computational efficiency was further evaluated in terms of model size, computational complexity, runtime, and memory usage. The proposed model contains 11,667 trainable parameters (0.0445 MB) and requires 5.1963 M MACs, with an average profiler-estimated 18.6094 ± 0.0251 M FLOPs. For a single sample, inference takes 15.45 ± 2.57 ms. Across 500 epochs, the total training time is 752.31 ± 46.85 s, with an average of 1.417 ± 0.089 s per training epoch and 0.0861 ± 0.0045 s per evaluation. Peak GPU memory usage is 412.71 ± 0.41 MB during training and 60.98 ± 10.06 MB during inference, and the combined parameter-plus-optimizer memory footprint is 0.1392 MB.

## 5 Discussion

In the current study, we emphasize evaluating two critical yet often overlooked attributes of Riemannian Geometry (RG) models for BCI deep learning frameworks: model stability and adaptability to small-sample data. These attributes are particularly vital given BCI’s primary application in rehabilitation, where the practical utility of devices is frequently constrained by repeated calibration requirements. The demonstrated effectiveness of RG models in motor decoding tasks establishes a foundational pathway toward calibration-free BCI systems. Should these models maintain robust performance under small-sample and noisy conditions, their practical value would be significantly enhanced.

The significant individual variability in EEG signals (Rahman et al., 2024), coupled with the limited availability of raw EEG data, presents major challenges for developing generalizable models suitable for a wide range of BCI applications (Li et al., 2025). This data bottleneck manifests primarily in two aspects. First, acquiring high-quality, large-scale, and well-annotated EEG data—particularly from specific patient groups such as stroke or spinal cord injury patients—is costly and time-consuming. Second, the inherent high dimensionality, low signal-to-noise ratio, and significant inter-subject/intra-subject variability of EEG signals make features learned by models difficult to transfer to new users or sessions (Zhong et al., 2024). Although domain adaptation (Lei et al., 2025; Liang et al., 2024) and domain generalization (Zhong et al., 2024) techniques attempt algorithmically to bridge the gap between different data distributions, their effectiveness is often constrained by the scale and quality of the source domain data. Therefore, persistently developing more robust and efficient model architectures and learning algorithms, while fundamentally addressing data scarcity, constitutes a key strategy for overcoming the BCI generalization bottleneck. The value of this study lies in establishing a large-scale cross-subject dataset. Designed with carefully crafted experimental paradigms to ensure ecological validity, this dataset systematically collected data from 227 healthy participants. It provides a vital platform for in-depth exploration of the nature of individual differences and evaluation of various domain adaptation and generalization techniques in real-world scenarios.

Meanwhile, our paper introduces, a classification algorithm of motion imagery based on a Riemannian space perturbation-aware dual-stream attention mechanism (PADA). In PADA architecture, the primary network focuses on the evolutionary patterns of features across temporal, frequency, and spatial domains, while the auxiliary network establishes cross-channel differential correlations through Riemannian metric learning. The two networks are fused using attention gating to integrate information effectively. Table 2 presents the results of within-subject classification tests using a 4-s time window. PADA achieved an average accuracy of 81.44% after 500 training epochs. Under this condition, five out of nine participants exceeded the 80% accuracy threshold. We compared the performance of our model on the BCI Competition IV-2a dataset with other deep learning architectures evaluated on the same dataset. For instance, the TCNet model proposed by (Ingolfsson et al., 2020), which employs temporal convolutional networks to capture long-range dependencies in EEG signals, and the MI-CAT model proposed by (Zhang et al., 2023), which utilizes the self-attention and cross-attention mechanisms of Transformer to obtain interactive features, achieved accuracies of 77.16 and 76.74%, respectively. Both results are approximately 4–5% lower than that of our model. Furthermore, compared to the WCSPAR model (Han et al., 2024), which solely incorporates Riemannian distance, and the MMDA model (Gao et al., 2022), which employs Riemannian alignment, our model constructs a more comprehensive Riemannian space representation by designing multiple Riemannian modules. In terms of accuracy, our model outperforms these two models by 4.51 and 3.51%, respectively.

It is noteworthy that the decoding accuracy of our model is not the highest among all models. For instance, the studies of (Altaheri et al., 2022; Liang et al., 2025) achieved a decoding accuracy of 85.27 and 87.65%, respectively. This may stem from our Riemannian geometry-based model placing greater emphasis on robustness and interference resistance, resulting in slightly lower accuracy—a characteristic inherent to this type of model. Crucially, the proposed model possesses the strongest interference resistance. This capability was validated by testing its recognition performance under various noise levels through the addition of noise.

The robustness advantages of our proposed PADA framework can be understood from two perspectives. First, the tangent space dynamic perturbation mechanism imposes geometrically constrained noise within the Log-Euclidean tangent space, exposing the model to a wide range of perturbations during the early stages of training. As training progresses, the perturbations are gradually annealed, and this curriculum-based strategy forces the network to learn invariance to local variations on the manifold. Second, the two-stream differential attention leverages the differential operation, utilizing the perturbed stream as a sensitivity probe. If a manifold connection exhibits significant changes under perturbation, the corresponding weight in the perturbed stream increases, and the connection is subsequently suppressed after the differential subtraction. Conversely, geometrically stable connections are preserved. This intrinsic soft-masking mechanism enables the model to self-supervisely identify and down-weight unreliable dependencies without requiring external noise labels. This design is directly validated by the experimental results in Table 7. Under 50% noise, PADA suffers only a 12.42% performance degradation, substantially lower than SecNet’s 20.15%, confirming the synergistic effectiveness of differential attention and geometry-aware perturbation.

Ablation study results further confirmed that the dual-stream differential fusion combined with the active perturbation mechanism significantly enhances model robustness by enriching feature representations (Table 8). Specifically, introducing the learnable amplifier alone (ratio 0.7) yields a 0.43% improvement, indicating that appropriate scaling of SPD features enhances nonlinear representation without compromising geometric stability. Adding Stream 1 (original manifold attention) on top of the amplifier improves accuracy to 78.16% (a 1.85% gain over baseline), confirming the effectiveness of manifold attention in capturing intrinsic geometric patterns. Further activating Stream 2 (perturbed tangent-space attention) raises accuracy to 79.03% (a 2.72% gain over baseline). This significant gain directly validates our core hypothesis: injecting diversity through tangent-space perturbation effectively enhances model robustness against spatiotemporal variability. Notably, the addition of Stream 2 alone contributes an extra 0.87% improvement, demonstrating the practical utility of the perturbed stream as a sensitivity indicator within the differential fusion strategy. Finally, applying Selective Euclidean Space Mapping (SEPM) achieves 81.44% accuracy, a total gain of 5.13% over baseline, indicating that this regularization mechanism effectively prevents overfitting by randomly dropping tangent-space features prior to classification. Compared to traditional multi-head attention modules (Altaheri et al., 2022) and single-stream attention mechanisms (Ma et al., 2024), this architecture is better suited for real-time applications in practical settings (Yue et al., 2026), demonstrating stronger practical relevance.

In addition to these encouraging results, we have retained several core design elements in our methodology to allow for further testing of the current benchmark when new motor imagery data becomes available or new methods emerge. For instance, unlike some prior studies (see (Altaheri et al., 2022; Liang et al., 2025)) which primarily focused on accuracy across different public datasets, we deliberately centered our research on robustness in practical application contexts. Consequently, this study emphasizes the model’s accuracy on small data samples to validate the generalization capability of the proposed technique. As shown in Table 4, our model achieves achieves up to 70.46% binary classification accuracy on the Classic-paradigm hand motor imagery task, and consistently outperforms baseline methods across all four independent binary tasks (hand/feet × Classic/CEP) on our CEP MI dataset. These findings provide critical guidance for optimizing experimental paradigms in practical BCI applications.

Although the main experiments were conducted under a subject-dependent within-subject protocol, we additionally performed LOSO cross-validation on the BCI IV-2a dataset to assess cross-subject generalization. The LOSO results indicate that further domain adaptation is still needed. Additionally, the model’s accuracy remains suboptimal. Future research should integrate prior knowledge of brain activity to refine feature extraction processes, thereby achieving more effective feature representation.

## 6 Conclusion

This study proposes an algorithm of motion imagery algorithm based on a perturbation-aware dual-stream attention mechanism in Riemannian space. By integrating convolutional neural networks with Riemannian geometry and leveraging spatial perturbation mechanisms alongside differential attention fusion, the proposed method effectively extracts spatial features from EEG signals, thereby enhancing algorithmic robustness. These advantages are demonstrated in two key aspects. Firstly, under extreme conditions with injected noise at 50% standard deviation, the model maintains 69.02% accuracy—a mere 12% performance decrease compared to the 20–40% degradation typically observed in existing models. Secondly, PADA achieves up to 70.46% accuracy on the classic hand MI task with 227 participants, reducing training by 94% compared to conventional methods (>200 trials). This breakthrough significantly shortens training cycles and streamlines experimental procedures, effectively addressing small-sample learning bottlenecks while providing innovative technical support for advancing non-invasive BCI practical implementation. Collectively, these contributions form a comprehensive solution to overcome BCI generalization barriers.

## Data Availability

The raw data supporting the conclusions of this article will be made available by the authors, without undue reservation.

## References

[ref1] Alimardani F. Boostani R. Blankertz B. (2017). Weighted spatial based geometric scheme as an efficient algorithm for analyzing single-trial EEGS to improve cue-based BCI classification. Neural Netw. 92, 69–76. doi: 10.1016/j.neunet.2017.02.014, 28385624

[ref2] Altaheri H. Muhammad G. Alsulaiman M. (2022). Physics-informed attention temporal convolutional network for EEG-based motor imagery classification. IEEE Trans. Ind. Inform. 19, 2249–2258. doi: 10.1109/tii.2022.3197419

[ref3] Ang K. K. Chin Z. Y. Zhang H. Guan C. (2008). “Filter Bank common spatial pattern (FBCSP) in brain-computer Interface,” in 2008 IEEE International Joint Conference on Neural Networks (IEEE World Congress on Computational Intelligence), (New York: IEEE).

[ref4] Arvaneh M. Guan C. Ang K. K. Quek H. C. (2011). “Spatially sparsed common spatial pattern to improve BCI performance,” in 2011 IEEE International Conference on Acoustics, Speech and Signal Processing (ICASSP), (New York: IEEE), 2412–2415.

[ref5] Bai Y. (2025). Application of motor imagery brain-computer interface training based on electroencephalogram in stroke rehabilitation. Rehabil. Med. 36, 75–81. doi: 10.3724/sp.j.1329.2026.02001

[ref6] Barachant A. Bonnet S. Congedo M. Jutten C. (2012). Multiclass brain–computer Interface classification by Riemannian geometry. IEEE Trans. Biomed. Eng. 59, 920–928. doi: 10.1109/TBME.2011.2172210, 22010143

[ref7] Chen C. S. Kuo E. J. Chen G. Y. Zhang X. Zhang F. (2026). A unified SPD token transformer framework for EEG classification: systematic comparison of geometric embeddings. arXiv 1:21521. doi: 10.48550/arXiv.2601.21521

[ref8] Chevallier S. Kalunga E. K. Barthélemy Q. Monacelli E. (2021). Review of Riemannian distances and divergences, applied to SSVEP-based BCI. Neuroinformatics 19, 93–106. doi: 10.1007/s12021-020-09473-9, 32562187

[ref9] Gao Y. Liu Y. She Q. Zhang J. (2022). Domain adaptive algorithm based on multi-manifold embedded distributed alignment for brain-computer interfaces. IEEE J. Biomed. Health Inform. 27, 296–307. doi: 10.1109/JBHI.2022.3218453, 36315544

[ref10] Han R. Li Z. Zhang Y. Meng X. Wang Z. Dong H. (2024). Weighted common spatial pattern based adaptation regularization for multi-source EEG time series. Comput. Electr. Eng. 120:109680. doi: 10.1016/j.compeleceng.2024.109680

[ref11] Hwaidi J. Ghanem M. C. (2026). Motor imagery EEG signal classification using minimally random convolutional kernel transform and hybrid deep learning. NeuroImage 328:121816. doi: 10.1016/j.neuroimage.2026.121816, 41719718

[ref12] Ingolfsson T. M. Hersche M. Wang X. Kobayashi N. Cavigelli L. Benini L. "EEG-TCNet: an accurate temporal convolutional network for embedded motor-imagery brain–machine interfaces," in 2020 IEEE International Conference on Systems, man, and Cybernetics (SMC), (2020), pp. 2958–2965 New York: IEEE.

[ref13] Jeannerod M. (2001). Neural simulation of action: a unifying mechanism for motor cognition. NeuroImage 14, S103–S109. doi: 10.1006/nimg.2001.0832, 11373140

[ref14] Jin J. Qu T. Xu R. Wang X. Cichocki A. (2023). Motor imagery EEG classification based on Riemannian sparse optimization and Dempster-Shafer fusion of multi-time-frequency patterns. IEEE Trans. Neural Syst. Rehabil. Eng. 31, 58–67. doi: 10.1109/TNSRE.2022.3217573, 36288214

[ref15] Lawhern V. J. Solon A. J. Waytowich N. R. Gordon S. M. Hung C. P. Lance B. J. (2018). EEGNet: a compact convolutional neural network for EEG-based brain–computer interfaces. J. Neural Eng. 15:056013. doi: 10.1088/1741-2552/aace8c, 29932424

[ref16] Lei C. Chen C. P. Chen C. L. P. Zhang T. (2025). Am-convbls: adaptive manifold convolutional broad learning system for cross-session and cross-subject emotion recognition. IEEE Trans. Affect. Comput. 16, 2788–2802. doi: 10.1109/taffc.2025.3565570

[ref17] Li J. Hao Y. Zhang W. Li X. Hu B. (2021). Effective connectivity based EEG revealing the inhibitory deficits for distracting stimuli in major depression disorders. IEEE Trans. Affect. Comput. 14, 694–705. doi: 10.1109/taffc.2021.3054953

[ref18] Li J. Shi J. Yu P. Yan X. Lin Y. (2025). Feature-aware domain invariant representation learning for EEG motor imagery decoding. Sci. Rep. 15:10664. doi: 10.1038/s41598-025-95178-5, 40148520 PMC11950222

[ref19] Liang W. Allison B. Z. Xu R. He X. Wang X. Cichocki A. . (2025). SecNet: a second order neural network for MI-EEG. Inf. Process. Manag. 62:104012. doi: 10.1016/j.ipm.2024.104012

[ref20] Liang Z. Zheng Z. Chen W. Pei Z. Wang J. Chen J. (2024). A novel deep transfer learning framew.ork integrating general and domain-specific features for EEG-based brain–computer interface. Biomed. Signal Process. Control 95:106311. doi: 10.1016/j.bspc.2024.106311

[ref21] Luo Y. Wang H. Chen Z. Wang J. Gan Z. Zhang L. . (2025). Integrating channel priors and shared pattern learning for enhanced motor imagery EEG classification. Neurocomputing 669:132532. doi: 10.1016/j.neucom.2025.132532

[ref22] Ma X. Chen W. Pei Z. Zhang Y. Chen J. (2024). Attention-based convolutional neural network with multi-modal temporal information fusion for motor imagery EEG decoding. Comput. Biol. Med. 175:108504. doi: 10.1016/j.compbiomed.2024.108504, 38701593

[ref23] Manivannan S. Lakshminarayan C. (2025). Geometry-aware deep congruence networks for manifold learning in cross-subject motor imagery. arXiv 1:18940. doi: 10.48550/arXiv.2511.18940

[ref24] Meng J. Wei Y. Mai X. Li S. Wang X. Luo R. . (2025). Paradigms and methods of noninvasive brain-computer interfaces in motor or communication assistance and rehabilitation: a systematic review. Med. Biol. Eng. Comput. 63, 2209–2233. doi: 10.1007/s11517-025-03340-y, 40059266

[ref25] Pan Y.-T. Chou J.-L. Wei C.-S. (2022). MAtt: a manifold attention network for EEG decoding. Adv. Neural Inf. Process. Syst. 35, 31116–31129. doi: 10.52202/068431-2256

[ref26] Pan L. Sun X. Wang K. Cao Y. Xu M. Ming D. (2025). Cross-session motor imagery-electroencephalography decoding with Riemannian spatial filtering and domain adaptation. Sheng Wu Yi Xue Gong Cheng Xue Za Zhi 42, 272–279. doi: 10.7507/1001-5515.202411035 PMC12035623 40288968

[ref27] Pan L. Wang K. Xu L. Sun X. Yi W. Xu M. . (2023). Riemannian geometric and ensemble learning for decoding cross-session motor imagery electroencephalography signals. J. Neural Eng. 20:066011. doi: 10.1088/1741-2552/ad0a01, 37931299

[ref28] Qu X. (2014). Age-related cognitive task effects on gait characteristics: do different working memory components make a difference. J. Neuroeng. Rehabil. 11:149. doi: 10.1186/1743-0003-11-149, 25348927 PMC4221663

[ref29] Rahman N. Khan D. M. Masroor K. Arshad M. Rafiq A. Fahim S. M. (2024). Advances in brain-computer interface for decoding speech imagery from EEG signals: a systematic review. Cogn. Neurodyn. 18, 3565–3583. doi: 10.1007/s11571-024-10167-0, 39712121 PMC11655741

[ref30] Schirrmeister R. T. Springenberg J. T. Fiederer L. D. J. Glasstetter M. Eggensperger K. Tangermann M. . (2017). Deep learning with convolutional neural networks for EEG decoding and visualization. Hum. Brain Mapp. 38, 5391–5420. doi: 10.1002/hbm.23730, 28782865 PMC5655781

[ref31] She Q. Chen T. Fang F. Zhang J. Gao Y. Zhang Y. (2023). Improved domain adaptation network based on Wasserstein distance for motor imagery EEG classification. IEEE Trans. Neural Syst. Rehabil. Eng. 31, 1137–1148. doi: 10.1109/TNSRE.2023.3241846, 37022366

[ref32] Shi Y. Jiang A. Zhong J. Li M. Zhu Y. (2024). Multiclass classification framework of motor imagery EEG by Riemannian geometry networks. IEEE J. Biomed. Health Inform. 29, 935–947. doi: 10.1109/jbhi.2024.3496757, 39527418

[ref33] Song Y. Zheng Q. Liu B. Gao X. (2022). EEG conformer: convolutional transformer for EEG decoding and visualization. IEEE Trans. Neural Syst. Rehabil. Eng. 31, 710–719. doi: 10.1109/TNSRE.2022.3230250, 37015413

[ref34] Srinivasan K. J. Periasamy P. Jayalaksshme Srinivasan K. Gunasekaran S. (2025). Motor imagery and motor execution: a narrative review of electroencephalographic (EEG) signatures, methodological consistency, and translational applications. Cureus 17:93011. doi: 10.7759/cureus.93011, 41141194 PMC12550537

[ref35] Tangermann M. Müller K.-R. Aertsen A. Birbaumer N. Braun C. Brunner C. . (2012). Review of the BCI competition IV. Front. Neurosci. 6:55. doi: 10.3389/fnins.2012.00055, 22811657 PMC3396284

[ref36] Tibermacine I. E. Russo S. Tibermacine A. Rabehi A. Nail B. Kadri K. . (2024). Riemannian geometry-based EEG approaches: a literature review. arXiv 1:20250. doi: 10.48550/arXiv.2407.20250

[ref37] Wang X. Yang W. Qi W. Wang Y. Ma X. Wang W. (2024). STaRNet: a spatio-temporal and Riemannian network for high-performance motor imagery decoding. Neural Netw. 178:106471. doi: 10.1016/j.neunet.2024.106471, 38945115

[ref38] Wilson D. Schirrmeister R. T. Gemein L. Ball T. (2025). Deep Riemannian networks for end-to-end EEG decoding. Imaging Neurosci. 3:511. doi: 10.1162/imag_a_00511 PMC12319850 40800883

[ref39] Xiong X. Su L. Guo J. Song T. Wang Y. Huang J. . (2025). Enhancing motor imagery decoding in brain–computer interfaces using Riemann tangent space mapping and cross frequency coupling. Biomed. Signal Process. Control 99:106797. doi: 10.1016/j.bspc.2024.106797

[ref40] Xue Q. Song Y. Wu H. Cheng Y. Pan H. (2024). Graph neural network based on brain inspired forward-forward mechanism for motor imagery classification in brain-computer interfaces. Front. Neurosci. 18:1309594. doi: 10.3389/fnins.2024.1309594, 38606308 PMC11008472

[ref41] Ying A. Lv J. Huang J. Wang T. Si P. Zhang J. . (2025). A feature fusion network with spatial-temporal-enhanced strategy for the motor imagery of force intensity variation. Front. Neurosci. 19:1591398. doi: 10.3389/fnins.2025.1591398, 40620352 PMC12226481

[ref42] Yue C. Ma Z. Xia Z. Zhang X. Zhang Y. Shen X. . (2026). LI-DSN: a layer-wise interactive dual-stream network for EEG decoding. arXiv 1:e01889. doi: 10.48550/arXiv.2604.01889

[ref43] Zhang D. Li H. Xie J. (2023). MI-CAT: a transformer-based domain adaptation network for motor imagery classification. Neural Netw. 165, 451–462. doi: 10.1016/j.neunet.2023.06.005, 37336030

[ref44] Zhang J. Li K. Yang B. Han X. (2023). Local and global convolutional transformer-based motor imagery EEG classification. Front. Neurosci. 17:1219988. doi: 10.3389/fnins.2023.1219988, 37662099 PMC10469791

[ref45] Zhao H. Zheng Q. Ma K. Li H. Zheng Y. (2020). Deep representation-based domain adaptation for nonstationary EEG classification. IEEE Trans. Neural Netw. Learn. Syst. 32, 535–545. doi: 10.1109/tnnls.2020.3010780, 32745012

[ref46] Zhong X.-C. Wang Q. Liu D. Chen Z. Liao J.-X. Sun J. . (2024). EEG-DG: a multi-source domain generalization framework for motor imagery EEG classification. IEEE J. Biomed. Health Inform. 29, 2484–2495. doi: 10.1109/jbhi.2024.3431230 39052465

[ref47] Zhou B. Wu X. Lv Z. Zhang L. Guo X. (2016). A fully automated trial selection method for optimization of motor imagery based brain-computer interface. PLoS One 11:e0162657. doi: 10.1371/journal.pone.0162657, 27631789 PMC5025076

[ref48] Zhuo F. Zhang X. Tang F. Yu Y. Liu L. (2024). Riemannian transfer learning based on log-Euclidean metric for EEG classification. Front. Neurosci. 18:1381572. doi: 10.3389/fnins.2024.1381572, 38872939 PMC11169813

