# Supplementary material for: PADA: perturbation-aware differential Riemannian attention for robust motor imagery decoding
Source: Front Neurosci. 2026 Sep 24;20:1943934. doi: 10.3389/fnins.2026.1943934 (PMC13646087; doi:10.3389/fnins.2026.1943934)
Supplement: Supplementary file 1 [file Data_sheet_1.docx]

**Supporting Information**

**PADA: Perturbation-Aware Differential Riemannian Attention for Robust Motor Imagery Decoding**

**Contents**

Appendix.1 Statistic analysis

Appendix.2 Cross-subject generalization analysis

Appendix.3 Robustness Analysis

Appendix.4 Frequency-specific contributions to MI discrimination

Appendix.5 Ablation Study

Fig. S3. Subject‑specific performance evaluation of the proposed model on the BCI IV‑2a dataset.

**Appendix.1 Statistic analysis**

**TABLE S1** indicates that PADA achieves statistically significant improvements over most competing methods under the subject-wise evaluation protocol. In particular, the gains over classical CSP-based methods and several deep learning baselines remain significant after Holm-Bonferroni correction, suggesting that PADA provides a robust advantage across subjects. By contrast, the improvements over TCNet and MI-CAT are positive but do not survive multiple-comparison correction, which implies that these recent models are closer competitors. ATCNet is the only baseline with a higher mean accuracy than PADA, but the paired difference is not statistically significant, indicating that PADA remains competitive but is not uniformly superior to every strong recent method.

TABLE S1

SUBJECT-WISE PAIRED WILCOXON SIGNED-RANK TEST RESULTS FOR PADA VERSUS BASELINE METHODS ON THE BCI COMPETITION IV-2A DATASET WITH HOLM-BONFERRONI CORRECTION.

| tBaseline | ΔAcc (%) | 95% CI | Corrected p | Effect size (r) |
| --- | --- | --- | --- | --- |
| SSCSP | +15.28 | [7.57, 22.98] | 0.0469 | 1.000 |
| FBCSP | +13.38 | [9.28, 17.49] | 0.0469 | 1.000 |
| ShallowConvNet | +9.23 | [5.82, 12.63] | 0.0469 | 1.000 |
| EEGNet | +4.97 | [1.99, 7.96] | 0.0469 | 0.956 |
| DRDA | +6.74 | [3.23, 10.26] | 0.0469 | 1.000 |
| DRDW | +3.84 | [1.45, 6.24] | 0.0469 | 0.956 |
| EEGConformer | +7.06 | [0.09, 14.04] | 0.0469 | 0.956 |
| EEG-TransNet | +8.83 | [2.80, 14.87] | 0.0469 | 1.000 |
| MAtt | +6.67 | [2.00, 11.35] | 0.0469 | 1.000 |
| MI-CAT | +4.63 | [0.23, 9.02] | 0.0781 | 0.833 |
| TCNet | +4.28 | [1.53, 7.03] | 0.0586 | 0.867 |
| ATCNet | -3.86 | [-12.38, 4.66] | 0.4961 | -0.289 |

**Appendix.2 Cross-subject generalization analysis**

To address the concern regarding cross-subject generalization, we conducted an additional leave-one-subject-out (LOSO) experiment on the BCI Competition IV-2a dataset. In each of the nine folds, data from one subject was held out as the unseen test set, one remaining subject was used for validation, and the other seven subjects were used for training, following the same preprocessing, training schedule, and random seed as the main experiments.

TABLE S2

PERFORMANCE COMPARISON ON THE BCI2A DATASET UNDER THE LOSO PROTOCOL.

| Model | Year | Cross-subject design | Accuracy(%) |
| --- | --- | --- | --- |
| CTNet [1] | 2024 | LOSO | 58.64±14.61 |
| DeepConvNet [2] | 2017 | LOSO | 60.15±12.71 |
| EEGNet [3] | 2018 | LOSO | 56.85±15.82 |
| Conformer [4] | 2022 | LOSO | 53.41±17.08 |
| **PADA(our)** | 2026 | LOSO | 39.54±9.62 |
| **PADA+C2(our)** | 2026 | LOSO | 59.69±16.70 |

As shown in TABLE S2, under the LOSO protocol, the proposed PADA model achieves an accuracy of 39.54%±9.62%, which is substantially lower than its subject-dependent performance. This performance drop is expected, as LOSO evaluation requires generalization to an entirely unseen target subject, exposing substantial inter-subject distribution shifts in EEG amplitude, spatial patterns, and ERD/ERS dynamics. This variability is further amplified on the BCI2a dataset by subject-specific anatomical differences, session non-stationarity, and trial-level noise. Since our current pipeline does not include an explicit target-subject calibration stage or a dedicated domain adaptation/alignment module, the learned representations remain partially subject-specific and do not transfer perfectly to unseen subjects. Notably, when augmented with the proposed C2 plugin [5], our model achieves a LOSO accuracy of 59.69% ± 16.70%, which is substantially above the 25% chance level for this four-class task and outperforms all compared baselines. This result suggests that the proposed component effectively improves cross-subject generalization, although subject-independent transfer remains the primary bottleneck for future work.

**Appendix.3 Robustness Analysis**

To investigate how PADA behaves under realistic recording degradations, we conducted three complementary robustness experiments that covered channel sparsity, channel missing/misalignment, and physiological artifacts. Specifically, we progressively reduced the number of retained channels to evaluate sensitivity to sparse montages, and we applied random dropout, region-specific dropout/noise, channel permutation, and local shift to approximate channel loss and electrode misalignment. We further injected EOG-, EMG-, and motion-like disturbances at different SNR levels to assess resistance to physiological interference. The results show that PADA degrades gradually under moderate perturbations, but the performance drops more sharply under severe channel identity disruption and motion-like artifacts. In particular, EOG-like artifacts are least harmful, whereas channel permutation and motion-like corruption are the most challenging conditions.

First, to simulate channel sparsity and electrode displacement, we evaluated channel selection, random dropout, region-specific dropout/noise, channel permutation, and local shift. As shown in **TABLE S3,** retaining only 16, 12, and 8 channels reduced accuracy from 81.44% to 64.00%, 51.08%, and 51.00%, respectively.

TABLE S3

CLASSIFICATION ACCURACY UNDER CHANNEL SPARSITY

| Retained channels | Accuracy (%) | Accuracy decrease（％） |
| --- | --- | --- |
| 22 | 81.44 | 0 |
| 16 | 64.00 | -17.44 |
| 12 | 51.08 | -30.36 |
| 8 | 51.00 | -30.44 |

For random dropout, we report the median, interquartile range (Q1-Q3), and full minimum–maximum range across multiple trials. The median accuracy dropped from 54% to 33% as the dropout probability *p* increased from 0.1 to 0.5. Sensorimotor-region dropout exhibited a counterintuitive peak at *p* = 0.3 (57%), which we speculate is due to the redundant information present in this region. Lateral-region dropout yielded consistently lower performance (17%-27%), suggesting limited contribution from lateral channels to the model. Notably, channel permutation caused the most severe degradation (35.69%), whereas local shift had a milder impact (68.67%). Overall, these results indicate that channel identity is more critical than spatial displacement for model performance; full statistical details are provided in **Fig. S1**.

**Fig. S1**. Performance summary statistics (%) under various channel perturbation scenarios. The table reports the maximum, third quartile (Q3), median, first quartile (Q1), and minimum accuracy across multiple trials for random dropout (a), sensorimotor-region dropout (b), lateral-region dropout (c), and misalignment proxies (d), each evaluated at perturbation probabilities *p* = 0.1, 0.3, and 0.5. Error bars represent the standard deviation.

Second, to mimic realistic physiological artifacts, we injected EOG-, EMG-, and motion-like disturbances at multiple SNR levels. EOG caused the mildest degradation (73.69% at 2 dB and 65.01% at –7 dB), whereas EMG and motion led to substantially larger drops—down to 59.03% and 50.96% at 2 dB, and 40.93% and 30.25% at –7 dB (see **Fig.S2**), respectively. Overall, these results indicate that PADA remains relatively stable under moderate perturbations but is more vulnerable to severe channel identity disruption and motion-like contamination.

**Fig. S2** Robustness under physiological artifiacts. Error bars represent the standard deviation.

**Appendix.4. Frequency-specific contributions to MI discrimination**

The proposed method utilizing the whole band achieved statistically significant improvements over single-band inputs in precision for the Tongue (*p* < 0.01) and Right-Hand (*p* < 0.05) tasks, as well as in recall for the Feet task (*p* < 0.05), with detailed p-values reported in the caption of **TABLE S4** and **TABLE S5.**

TABLE S4

Precision Difference Analysis

| classification | Friedman p | Overall difference | Rank Mean Trend（Whole / Alpha / Beta） | conclusion |
| --- | --- | --- | --- | --- |
| Left-Hand | 0.36788 | non-significant | 2.33 / 1.67 / 2.00 |  |
| Right-Hand | 0.04979 | significant | 2.67 / 1.67 / 1.67 |  |
| Feet | 0.45943 | non-significant | 2.33 / 1.78 / 1.89 |  |
| Tongue | 0.00483 | significant | 2.44 / 1.11 / 2.44 | Whole > Alpha，Beta > Alpha |

TABLE S5

Recall Difference Analysis

| classification | Friedman p | Overall difference | Rank Mean Trend (Whole/ Alpha/Beta) | conclusion |
| --- | --- | --- | --- | --- |
| Left-Hand | 0.45943 | non-significant | 2.22 / 1.67 / 2.11 |  |
| Right-Hand | 0.12451 | non-significant | 2.56 / 1.72 / 1.72 |  |
| Feet | 0.04455 | significant | 2.67 / 1.56 / 1.78 | Whole > Alpha |
| Tongue | 0.97260 | non-significant | 1.94 / 2.06 / 2.00 |  |

**Appendix.5 Ablation Study**

We added a more comprehensive component-wise ablation study on BCI Competition IV-2a to isolate the contribution of each key module, including the learnable amplifier, differential attention, perturbation module, SEPM, and manifold mapping. As shown in **TABLE S6**, the full model achieves 81.44% accuracy, while removing the amplifier, differential attention, perturbation, SEPM, and manifold mapping reduces the accuracy to 77.55%, 76.93%, 76.97%, 77.20%, and 76.47%, respectively. These results demonstrate that each component contributes to the final performance and provide a clearer understanding of their respective roles. We have updated the corresponding text and table in the Experimental Results section accordingly.

TABLE S6

COMPONENT-WISE ABLATION STUDY RESULTS ON THE BCI COMPETITION IV-2A DATASET.

| Variant | Amp. Ratio | First-stream | Second-stream | SEPM | Manifold mapping | Acc. (%) |
| --- | --- | --- | --- | --- | --- | --- |
| Full | 0.7 | √ | √ | √ | √ | **81.44** |
| w/o amplifier |  |  | √ | √ | √ | 77.55 |
| w/o differential attention | 0.7 | √ |  | √ | √ | 76.93 |
| w/o perturbation | 0.7 | √ | √ | √ | √ | 76.97 |
| w/o SEPM | 0.7 | √ | √ |  | √ | 77.20 |
| w/o manifold mapping |  |  | √ | √ | √ | 76.47 |

**Fig. S3.** Subject‑specific performance evaluation of the proposed model on the BCI IV‑2a dataset. The bar chart reports accuracy, precision, recall, and F1 score for each of the nine subjects (A01–A09). Each metric is color‑coded for clarity.Error bars represent the standard deviation.

**Reference**

[1] Z. Li, Y. Wang, et al., “CTNet: A convolutional transformer network for EEG-based motor imagery classification,” *IEEE Transactions on Neural Systems and Rehabilitation Engineering*, vol. 32, pp. 1234–1245, 2024.

[2] R. T. Schirrmeister, J. T. Springenberg et al., "Deep learning with convolutional neural networks for EEG decoding and visualization," *Human Brain Mapping*, vol. 38, no. 11, pp. 5391-5420, 2017.

[3] V. J. Lawhern, A. J. Solon et al., "EEGNet: a compact convolutional neural network for EEG-based brain–computer interfaces," *Journal of neural engineering*, vol. 15, no. 5, p. 056013, 2018.

[4]Y. Song, Q. Zheng et al., "EEG conformer: Convolutional transformer for EEG decoding and visualization," *IEEE Transactions on Neural Systems and Rehabilitation Engineering*, vol. 31, pp. 710-719, 2022.

[5] J. Li, H.Q *et al.*, "Group-Equivariant Riemannian Network with Hemispheric Mirror Symmetry Prior for Robust Motor Imagery Decoding, " in *2026 The 19th International Conference on Advanced Computer Theory and Engineering (ICACTE 2026)*, 2026, IEEE.
